# Multivariate Characterization of Gait Variability Under Simulated Ship Roll Motion

**DOI:** 10.3390/bioengineering13091039

**Published:** 2026-09-07

**Authors:** Namwoong Kim, Minsu Song, Jungyeon Choi

**Affiliations:** 1 Division of Health and Kinesiology, Incheon National University, Incheon 22012, Republic of Korea; nwkim@inu.ac.kr; 2 Department of Human Movement Science, Incheon National University, Incheon 22012, Republic of Korea; 3 Sports Science Institute, Incheon National University, Incheon 22012, Republic of Korea; 4 Sports Functional Disability Institute, Incheon National University, Incheon 22012, Republic of Korea; 5 Department of Economics, Chosun University, Gwangju 61452, Republic of Korea; smssms22342@naver.com; 6 Department of Computer Science and Statistics, Chosun University, Gwangju 61452, Republic of Korea

**Keywords:** gait variability, ship roll motion, principal component analysis, density-based clustering, inter-individual variability, musculoskeletal simulation, maritime occupational safety

## Abstract

Walking on a ship requires continuous adaptation to a support surface that changes orientation with vessel motion. However, conventional group-level and joint-by-joint analyses may not characterize the coordinated structure of this adaptation or the heterogeneity of individual responses. This secondary analysis examined multivariate lower-limb joint-angle variability during simulated ship roll in 30 healthy adults completing nine 2-min walking trials across roll amplitudes of 0–20° and roll periods of 6 and 12 s. OpenSim-derived joint angles were summarized as 28 trial-level variability features. Principal component analysis, *k*-means clustering, hierarchical density-based spatial clustering of applications with noise (HDBSCAN), and generalized estimating equations were applied. Five principal components explained 78.1% of the total variance, with PC1 alone explaining 53.4% and representing a coordinated increase in lower limb joint angle variability, particularly in the sagittal-plane. *k*-means derived operational partition classified trials into low- and high-variability groups. The odds of high-variability group membership increased 1.48-fold per 1° increase in roll amplitude (*p* < 0.001), whereas roll period was not significantly associated with group membership (*p* = 0.704). HDBSCAN showed weak density separation (DBCV = 0.061) and substantial participant-level heterogeneity. These findings highlight the potential value of individualized roll-sensitivity assessment and provide a biomechanical basis for future studies of shipboard gait safety and fall-risk assessment.

## 1. Introduction

Walking aboard a ship differs fundamentally from terrestrial locomotion because the support surface continuously translates and rotates beneath the body. This distinction has direct implications for maritime occupational safety. Slips, trips, and falls remain an important source of non-fatal injuries in seafaring, motivating a better understanding of gait adaptation under vessel motion [1,2]. Among the six degrees of vessel motion, roll is particularly relevant to human balance because it periodically changes the mediolateral inclination of the deck relative to gravity. Consequently, the relationship between the body’s center of mass and its base of support is repeatedly disturbed even when an individual attempts to walk in a straight line. When the resulting perturbation exceeds the capacity for postural correction or compensatory stepping, the ongoing task may be interrupted. Such events have been described as motion-induced interruptions and have long been used to relate vessel motion to crew operability and performance [3,4]. Shipboard gait therefore requires continuous biomechanical adaptation to a dynamically changing support surface, raising the question of how this adaptation is organized across the lower-limb joints.

Walter et al. [5] demonstrated that vessel motion alters both the perception and control of locomotion. Experienced seafarers adjust their judgments of walkable paths according to the changing dynamics of the body–ship system, suggesting that locomotor affordances are recalibrated under maritime motion [5]. Controlled simulation studies have further shown that increasing roll motion changes step timing, lateral center-of-mass displacement, and the margin of stability, indicating the disruption of gait regulation and dynamic balance [6]. A subsequent kinematic study using the same computer-assisted rehabilitation environment (CAREN) dataset examined lower-limb maximum and minimum joint angles and their cycle-to-cycle standard deviations through repeated-measures analysis [7]. That study showed that joint-angle variability increased systematically with roll amplitude, with particularly large effects in the sagittal-plane at the knee, hip, and ankle, where partial η^2^ values ranged from approximately 0.83 to 0.92. Nevertheless, the previous joint-by-joint analysis could not determine whether these changes reflected independent responses at individual joints or a coordinated adaptation involving the entire lower-limb chain. It also could not determine whether the group-average increase represents a broadly shared response or conceals individuals with markedly different sensitivities to roll motion.

This limitation is partly attributable to the conventional use of discrete-parameter and univariate analyses in gait biomechanics. Joint-angle waveforms are often reduced to a few discrete measures, such as maximum, minimum, or range-of-motion values, which are then analyzed separately. However, these features are strongly correlated because movements of the hips, knees, ankles, and subtalar joints are mechanically linked and coordinatively controlled. Analyzing each variable independently can therefore fragment a multijoint response, increase the burden of multiple comparisons, and obscure covariance across joints and anatomical planes. Principal component analysis provides an alternative framework by transforming correlated biomechanical variables into a smaller set of mutually orthogonal components while retaining most of the original information. Principal component analysis (PCA) has been widely used to characterize coordination in human gait and can reveal movement patterns that may not be apparent when biomechanical variables are analyzed separately [8,9,10]. From a biomechanical perspective, a key question is whether roll-induced variability reflects a shared multijoint pattern across the lower limb or several joint-specific patterns. Characterizing this structure may clarify how lower-limb variability is organized in response to roll perturbations and provide interpretable measures of roll-induced changes in gait variability.

A further limitation of population-level analysis is its inability to identify individuals whose responses depart from the dominant group pattern. A group-average response curve may underestimate sensitive individuals who exhibit atypical gait variability at smaller roll amplitudes while simultaneously overestimating vulnerability in individuals who remain stable at greater amplitudes. Unsupervised clustering has been used to identify clinically and biomechanically meaningful gait phenotypes in populations with cerebral palsy, hereditary spastic paraplegia, knee osteoarthritis, and other movement disorders [11,12,13]. In the present context, centroid-based *k*-means clustering can provide a practical partition of gait trials into low- and high-variability profiles. However, *k*-means assigns every observation to a cluster even when the underlying distribution is continuous and lacks naturally separated groups. A hierarchical density-based spatial clustering of applications with noise (HDBSCAN) offers a complementary density-based approach because it can identify a dominant dense structure while leaving low-density observations unassigned rather than forcing them into a predefined category [14]. These low-density observations may indicate responses that depart from the dominant multivariate pattern. They should not be regarded automatically as pathological gait or direct evidence of fall risk, but their concentration within particular participants may reveal meaningful inter-individual response heterogeneity that cannot be identified from group means alone.

Accordingly, the primary purpose of this secondary analysis was to determine whether gait variability across multiple lower-limb joints during simulated ship-roll walking could be represented by a shared low-dimensional structure. The resulting representation was used to examine the associations of roll amplitude and period with variability-profile membership and to assess whether the apparent profiles reflected a distinctly clustered or predominantly continuous distribution. Participant-level departures from the dominant multivariate response were also explored.

## 2. Materials and Methods

### 2.1. Data Source

This study was a secondary analysis of kinematic data collected in a previous gait experiment conducted under simulated ship-roll conditions [6]. The original study recruited healthy adults aged 19–55 years. Participants were excluded if they had (i) a history of major lower-extremity injury or surgery; (ii) a known cardiovascular condition that made exercise unsafe; (iii) a history of dizziness associated with a vestibular disorder, including Ménière’s disease or vertigo; or (iv) difficulty walking in an unstable moving environment. The final sample comprised 30 participants (20 men and 10 women; age, 30.3 ± 6.1 years; observed range, 21–39 years). All participants provided written informed consent before data collection, and the original protocol was approved by the Institutional Review Board of the University of Nebraska Medical Center (IRB No. 141-21-EP). Each participant walked for 2 min per condition on a split-belt treadmill integrated into the CAREN (Motek, Amsterdam, The Netherlands) [15].

The experimental conditions comprised a baseline condition with no rolling (0°) and roll conditions of 5°, 10°, 15°, and 20°. Each roll amplitude was applied under a slow roll condition with a 12 s period and a fast roll condition with a 6 s period, yielding a total of nine gait trials per participant. After the baseline condition, the eight roll conditions were administered in random order to minimize learning effects. The roll motion was implemented within a maximum range of 20° using the six-degrees-of-freedom (6-DOF) motion platform of the CAREN (Table 1).

Whole-body motion during gait was recorded at 100 Hz using a three-dimensional motion-capture system consisting of ten cameras (Vicon Motion Systems Ltd., Oxford, UK) [16]. Following the Vicon Plug-in Gait full-body model, 37 reflective markers were attached to the head, trunk, upper limbs, pelvis, and lower limbs. Before data collection, the Xsens MVN Awinda system was calibrated using the manufacturer-recommended N-pose procedure in Xsens MVN software version 2021.0.1 (Xsens, Enschede, the Netherlands), and data were sampled at 100 Hz. Seven wireless inertial measurement unit (IMU) sensors (Xsens, Enschede, the Netherlands) were attached to the pelvis and bilateral thighs, shanks, and feet using elastic straps to record triaxial acceleration [6]. Sensor placement was standardized using anatomical landmarks, and sensors were mounted consistently according to the Xsens coordinate convention. The longitudinal axis of the thigh and shank sensors was aligned with the long axis of each segment, whereas that of the foot sensors was aligned from the heel toward the toes. Sensor connectivity and synchronization were verified before each trial. For temporal segmentation, the vertical acceleration signal recorded by the pelvis IMU was used to identify heel-strike events using a peak-detection procedure. The IMU signals were not used to calculate the joint angles analyzed in this study, which were derived from the marker trajectories through OpenSim inverse kinematics.

Joint angles were computed from the marker trajectories through musculoskeletal simulation (OpenSim 4.6) [17]. The full-body musculoskeletal model of Rajagopal et al., which has 37 degrees of freedom, was used as the base model [18]. One additional degree of freedom was introduced to compute the frontal-plane motion of the knee (knee abduction). After the model was scaled to each participant’s body segments, an inverse kinematics analysis was performed to obtain the joint-angle time series. Both scaling and inverse kinematics used an optimization procedure that minimizes the distance between the experimental markers and the virtual markers of the model.

### 2.2. Data Preprocessing

Heel-strike events were identified by applying a peak-detection algorithm to the vertical acceleration signal recorded by the pelvis IMU, with acceleration peaks corresponding to heel strikes [6,19]. Each gait cycle was defined as the interval from a heel strike of one foot to the next heel strike of the same foot. The joint-angle time series were segmented into gait cycles based on this definition, and the maximum and minimum values of each joint angle were extracted from each cycle. For each trial, the standard deviations of these segment-specific maxima and minima were then calculated to quantify cycle-to-cycle joint-angle variability.

A total of 14 joint angles were included. The channels consisted of the bilateral hip flexion, hip ad/abduction, hip rotation, knee flexion, knee ad/abduction, ankle (dorsi/plantarflexion), and subtalar angles. For each channel, the SD of the maximum angles and the SD of the minimum angles were derived, producing 28 variability features per trial (e.g., *knee_angle_l_max_sd*). The final feature matrix had a size of 270 trials × 28 features. All features were standardized to z-scores prior to cluster analysis.

### 2.3. Dimensionality Reduction

PCA was applied to the standardized 270 × 28 feature matrix to characterize the shared structure of multijoint gait variability and obtain a lower-dimensional representation for subsequent cluster analysis. The 28 trial-level variability features were derived from multiple bilateral lower-limb joints, and many of these features were correlated. PCA transforms correlated features into orthogonal principal components ordered according to the amount of variance they explain, allowing the dominant patterns of covariation across the lower limb to be summarized with fewer dimensions. PCA has similarly been used to characterize coordinative structures and reduce the dimensionality of biomechanical data [8,9].

The smallest number of components whose cumulative explained variance exceeded the 75% reference criterion was five (PC1–PC5), which collectively explained 78.1% of the total variance. The same PC1–PC5 score matrix was used as the common input for both *k*-means and HDBSCAN, allowing the centroid-based and density-based clustering results to be compared within an identical feature representation.

For interpretation, the principal-component coefficients—the elements of the component eigenvectors—were examined. The absolute magnitude of each coefficient indicates the weight of the corresponding feature in defining a component, whereas the relative signs indicate whether features vary in the same or opposite directions along that component. In the fitted PCA solution, all PC1 coefficients were positive; accordingly, larger PC1 scores represented greater overall multijoint variability. No post-estimation sign reversal was applied. For PC2–PC5, only the relative contrasts among features, rather than the absolute positive or negative orientation of each component, were interpreted.

Because the 270 trials comprised nine repeated observations from each of the 30 participants, they were not treated as statistically independent observations when assessing PCA stability. A participant-level bootstrap analysis with 1000 resamples was therefore performed to evaluate the sensitivity of the PCA solution to participant-level sampling variation. In each resample, 30 participants were sampled with replacement, and all nine trials from each selected participant were retained, thereby preserving the within-participant repeated-measures structure. The 28 gait-variability variables were re-standardized within each bootstrap sample, and PCA was refitted retaining five components.

Two aspects of PCA stability were evaluated. The explained variance ratio (EVR), representing the proportion of total variance explained by each ranked component, was used to assess the consistency of its variance contribution. For each component rank, the median and 2.5th–97.5th percentiles of the EVR were calculated; values close to the original EVR with narrow intervals indicated greater consistency. The reproducibility of the component loading patterns was assessed using Tucker’s congruence coefficient [20]. Because component order and sign may vary across bootstrap samples, the bootstrap components were matched one-to-one to the original components using absolute congruence values. For each original component, the median and 2.5th–97.5th percentiles were calculated, with values closer to 1 and narrower intervals indicating greater loading-pattern reproducibility.

### 2.4. Cluster Analysis

The rationale for applying two algorithms based on different principles to the same PC score space (PC1–PC5) was as follows. *k*-means assigns every observation to one of k clusters and thus shows how the condition-dependent responses are arranged at the population level. HDBSCAN, in contrast, can leave low-density observations unassigned as outliers, and can therefore identify observations that depart from the dominant density structure.

#### 2.4.1. *k*-Means (Centroid-Based)

*k*-means partitions all observations into a predefined number of clusters by minimizing the within-cluster sum of squares, assigning every trial to the nearest centroid. It therefore provides a complete, population-level partition of the variability space in which each trial is assigned to a profile, making it suitable for describing how condition-dependent responses are distributed across the whole sample. The number of clusters was searched over *k* = 2–8. To reduce sensitivity to initialization, the *k*-means ++ seeding method was used [21], and the model was run with multiple random initializations, retaining the solution with the lowest within-cluster sum of squares. The optimal number of clusters was selected using two internal validity indices with complementary properties: the Silhouette coefficient, which quantifies how well each observation fits its own cluster relative to the nearest neighboring cluster (larger values indicate better separation), and the Davies–Bouldin index, which measures the average similarity between each cluster and its most similar cluster (smaller values indicate better clustering) [22]. Both indices were evaluated across the candidate values of *k*, and the solution that jointly optimized them was retained as the main partition.

#### 2.4.2. HDBSCAN (Density-Based)

HDBSCAN identifies clusters as contiguous regions of high-density, and unlike *k*-means, does not force every observation into a cluster; observations that lie in low-density regions are left unassigned as noise [14]. This property makes it suitable for detecting trials that depart from the dominant density structure rather than imposing a fixed partition on the whole sample. The hyperparameters were selected through a grid search that maximized the density-based clustering validation (DBCV) index [23], an internal criterion designed specifically for density-based solutions. The search ranges were as follows:

- *min_cluster_size* ∈ [2, 25];
- *min_samples* ∈ [1, 15];
- *cluster_selection_epsilon* ∈ [0, 2.0] (17 values);
- metric = ‘euclidean’.

The optimum was obtained at *min_cluster_size* = 6, *min_samples* = 2, and *cluster_selection_epsilon* = 0.0 (DBCV = 0.061), yielding four clusters and a set of outliers. Trials labeled as outliers (−1) were interpreted not as observations belonging to a separate cluster, but as observations that departed from the dominant density structure.

### 2.5. Statistical Analysis

To test the effects of roll amplitude and roll period on membership in the high-variability profile, a logistic regression model was used because the outcome was binary. However, each participant contributed nine trials, so the observations were not independent. To account for this within-participant correlation, the logistic model was estimated within GEE [24,25], which extends generalized linear models to correlated (repeated-measures) data and yields population-averaged effect estimates [26]. The participant was specified as the clustering unit, and an exchangeable working correlation structure with robust sandwich standard errors was applied.

The dependent variable was membership in the k-means high-variability profile (1 = high-variability, 0 = low-variability). Roll amplitude was entered as a continuous variable in degrees (°), and roll period was coded as a binary variable (0 = slow, 1 = fast). The following models were fitted:

logit(Pr[Y_ij_ = 1]) = β_0_ + β_1_Amplitude_ij_ (1a)

logit(Pr[Y_ij_ = 1]) = β_0_ + β_1_Amplitude_ij_ (1b)

logit(Pr[Y_ij_ = 1]) = β_0_ + β_1_Amplitude*_ij_* + β_2_Period_ij_ (2)

logit(Pr[Y_ij_ = 1]) = β_0_ + β_1_Amplitude^C^_ij_+ β_2_Period_ij_ + β_3_(Amplitude^C^_ij_ × Period_ij_) (3)

Amplitude^C^_ij_ = Amplitude_ij_-10° (4)

where Y_ij_ is the binary indicator of whether the *j*-th trial of participant *i* belongs to the high-variability profile (1 = high, 0 = low); Pr[Y_ij_ = 1] is the probability of high-variability membership; Amplitude_ij_ is the roll amplitude of that trial in degrees; and Period_ij_ is the roll period (0 = slow, 1 = fast). β_0_ is the intercept (the log-odds when all predictors equal zero); β_1_ is the change in log-odds per 1° increase in roll amplitude; β_2_ is the difference in log-odds between the fast and slow conditions; and β_3_ is the amplitude × period interaction, representing the extent to which the amplitude effect differs between the two period conditions. Equation (1a), fitted to all 270 trials, estimated the association between roll amplitude and high-variability membership. Equation (2) additionally included roll period adjusted for amplitude, and Equation (3) added the roll amplitude × roll period interaction. Because roll period is undefined at 0° (no rolling occurred), that condition was excluded from Equations (2) and (3), which were therefore fitted to 240 trials. Equation (1b), an amplitude-only model, was additionally fitted to the same 240 trials to provide a comparison for Equation (2). In Equation (3), roll amplitude was centered at 10° (Equation (4)). The 10° condition was selected as an observed reference level, allowing the roll-period coefficient to represent the fast-versus-slow difference at 10° rather than at 0°, where roll period was undefined.

For each model, the regression coefficients were estimated, and the significance of each coefficient was assessed with a Wald test (z = β/SE); a significant coefficient indicated that the corresponding predictor was associated with the odds of high-variability membership. The amplitude coefficient β_1_ represents the change in log-odds per 1° increase in roll amplitude, and the corresponding odds ratios and 95% confidence intervals were reported per 1° increase. Coefficients were interpreted as odds ratios, with values greater than 1 indicating increased odds of membership in the high-variability profile, values less than 1 indicating decreased odds, and a value of 1 indicating no association. Statistical significance was set at α = 0.05.

### 2.6. Exploratory Participant-Level Analysis of HDBSCAN Outliers

To examine the concentration of HDBSCAN outlier labels within participants, the number and proportion of each participant’s nine trials labeled as outliers were calculated. The proportion of trials labeled as outliers was also summarized separately for each roll-amplitude condition. For descriptive group-level summarization, participants with an outlier rate greater than 0.50—that is, at least five out of nine trials labeled as outliers—were operationally classified as the frequent-outlier group, and the remaining participants were classified as the comparison group. This post hoc cutoff was not intended to represent a clinical or safety-related threshold.

To characterize the multivariate patterns associated with frequent outlier labeling, the scores for each retained PC were averaged across the nine trials within each participant. Differences in participant-level mean PC scores between the frequent-outlier and comparison groups were summarized using Cohen’s d, calculated as the difference between the group means divided by the pooled participant-level standard deviation. For descriptive interpretation, absolute Cohen’s d values below 0.20 were considered below the small-effect benchmark, values from 0.20 to <0.50 were classified as small, values from 0.50 to <0.80 as medium, and values ≥ 0.80 as large [27]. The sign indicated the direction of the group difference.

To describe the individual multivariate profiles of the frequent-outlier participants, each participant’s mean score for each PC was standardized using the mean and sample standard deviation of the corresponding participant-level scores across all 30 participants. Thus, a z-score of 0 represented the overall participant mean, and a larger absolute z-score represented a more extreme position within the present sample. Because the group classification and PC scores were derived from the same PCA–HDBSCAN analysis, all participant-level comparisons were considered exploratory and descriptive. No hypothesis testing was performed for these comparisons.

## 3. Results

### 3.1. Principal Component Analysis

**Figure 1:**
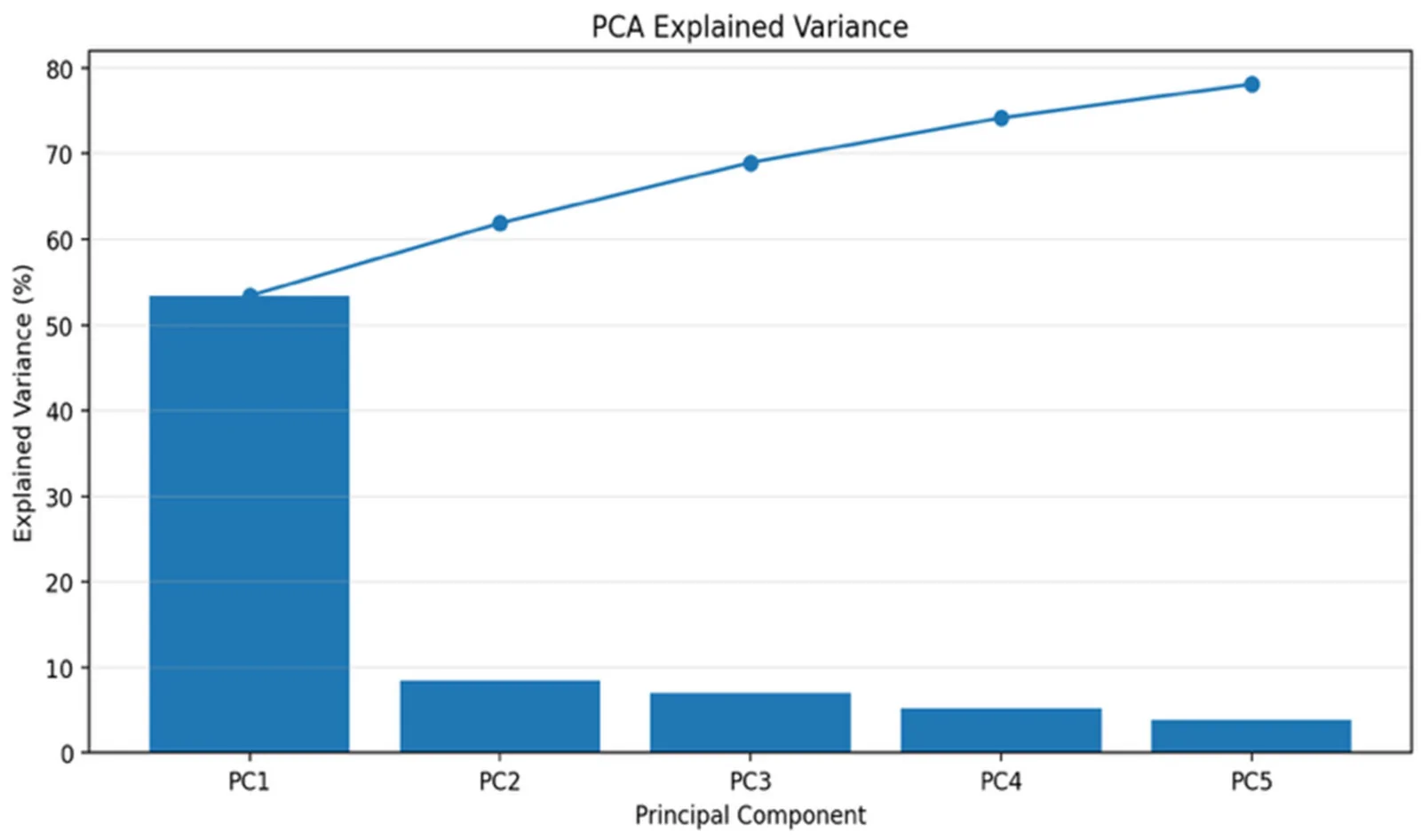
Explained variance of the principal components.

The explained variance of each principal component was 53.4% for PC1, 8.5% for PC2, 7.1% for PC3, 5.2% for PC4, and 3.9% for PC5. PC1–PC5 were retained as the minimum number of components satisfying the pre-specified cumulative explained variance criterion of 75%, with a cumulative explained variance of 78.1% (Figure 1 and Table 2). PC1 alone explained 53.4% of the total variance, whereas the explained variance of PC2 was 8.5%, less than one-sixth that of PC1. Beyond PC2, the explained variance decreased gradually and no clear elbow appeared. Thus, the covariance structure of the 28 variability features consisted of one dominant axis and many small axes.

**Figure 2:**
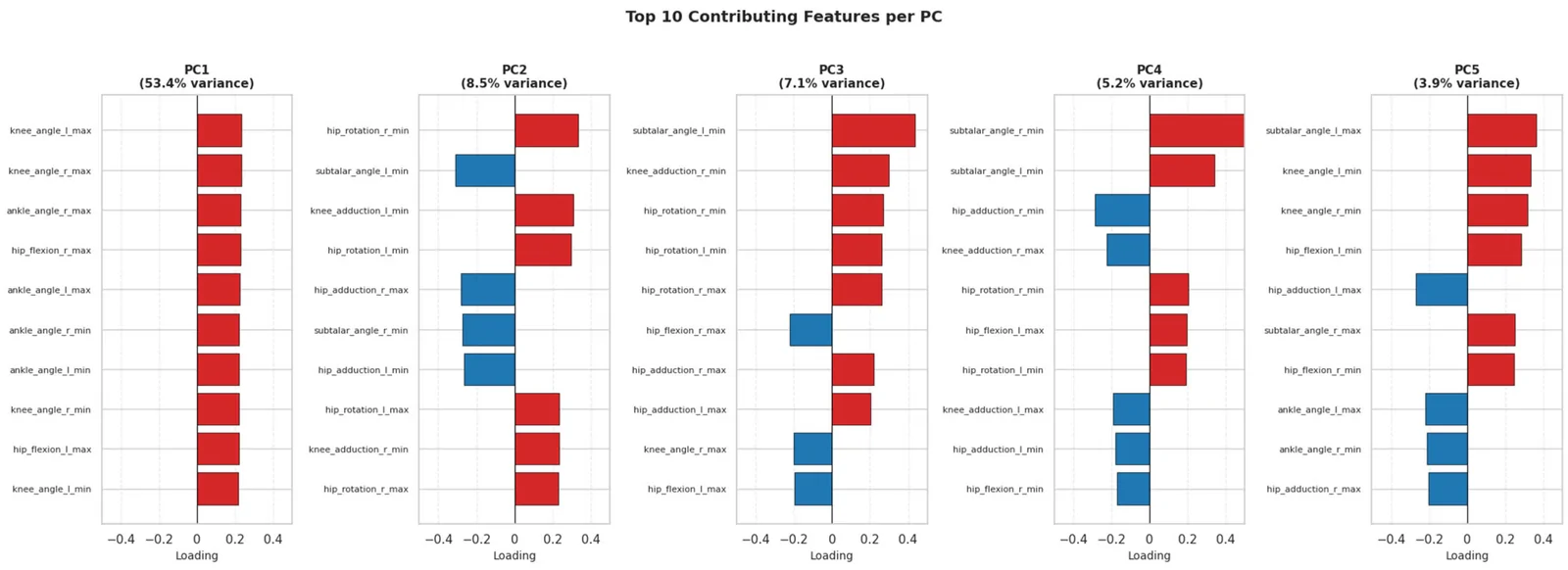
The ten features with the largest loadings for each principal component (PC1–PC5). Positive and negative loadings are shown in contrasting colors.

The PC coefficients are the elements of the eigenvectors that define each principal component and represent the weight of each original feature in that component. Features with the same sign co-vary in the same direction, and the larger the absolute value of the coefficient, the greater the contribution. The top ten weighted features of each PC are shown in Figure 2.

#### 3.1.1. PC1

All 28 coefficients of PC1 had the same positive sign (+). The fact that all coefficients were positive indicated that PC1 was not an axis that contrasted one joint group with another. A trial with a larger PC1 score had large joint-angle SDs across the whole lower limb simultaneously. PC1 therefore represented the overall magnitude of variability rather than its distribution. Moreover, the ten features with the largest coefficients were all the SDs of the sagittal-plane flexion/extension maximum and minimum angles of the bilateral knees, ankles, and hips, with a range of 0.217–0.234 (Table 3).

#### 3.1.2. PC2–PC5

Unlike PC1, the coefficients of PC2–PC5 contained a mixture of positive and negative signs, indicating contrasts among joint groups rather than overall variability magnitude. In the fitted PCA solution, PC2 primarily contrasted transverse- and frontal-plane variability, PC3 contrasted non-sagittal and sagittal variability, PC4 was dominated by subtalar variability, and PC5 contrasted minimum- and maximum-angle variability (Figure 2). Each of these components explained less than 9% of the total variance. PC1–PC5 were retained as the common input for clustering because they collectively satisfied the prespecified 75% cumulative explained-variance criterion; however, the joint- and plane-specific interpretations of PC2–PC5 were treated as exploratory.

#### 3.1.3. Participant-Level Bootstrap Stability

The participant-level bootstrap analysis showed that the PC1 loading structure was highly reproducible, whereas its EVR showed some variation across resamples (Table 4). The original EVR of PC1 was 0.5340, and its bootstrap median was 0.5420 (2.5th–97.5th percentile: 0.4979–0.5832). The median differed from the original value by 1.5%, while the percentile limits corresponded to relative deviations of −6.8% and +9.2%. The median absolute Tucker congruence coefficient for PC1 was 0.9971 (0.9917–0.9991), indicating highly reproducible loading patterns. For PC2–PC5, the congruence coefficients were smaller and showed substantially wider percentile intervals. Thus, the bootstrap analysis strongly supported the reproducibility of the dominant PC1 loading pattern, whereas the component-specific loading patterns of PC2–PC5 were less stable and should be interpreted with caution.

### 3.2. k-Means Clustering: Two Variability Profiles

**Figure 3:**
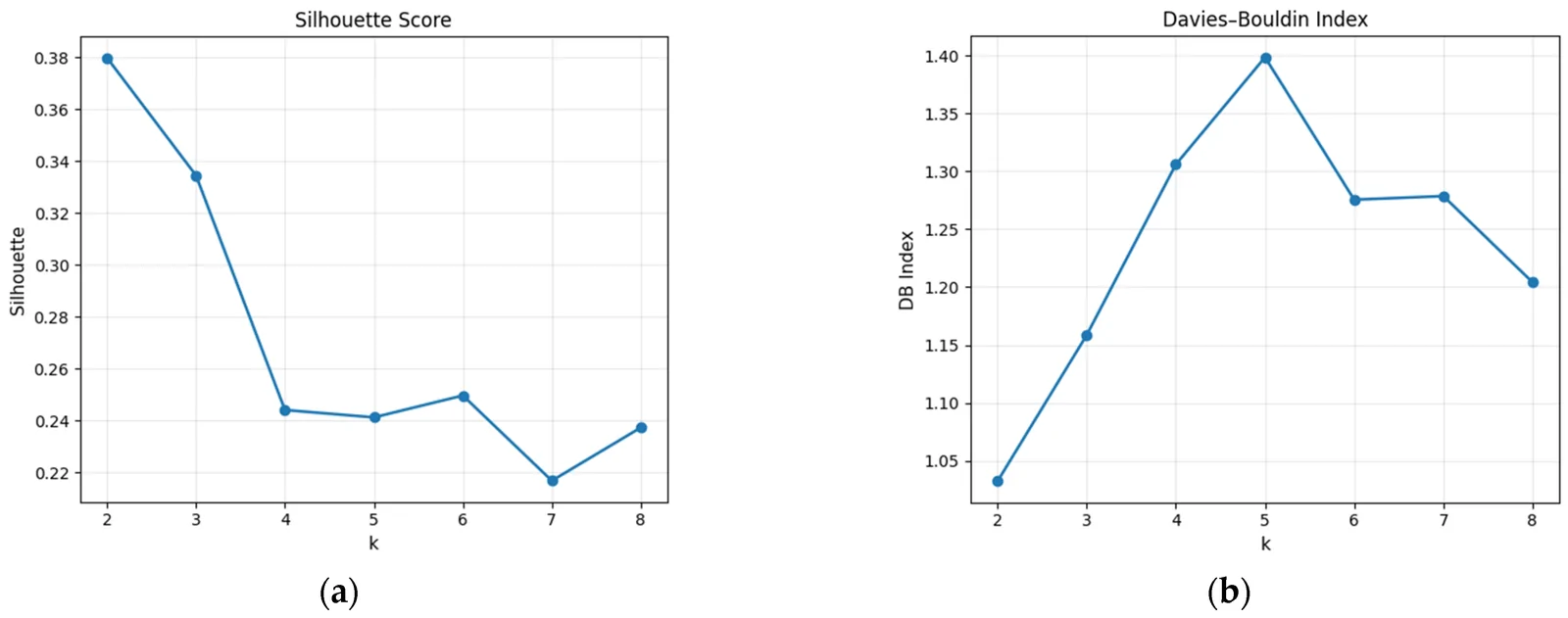
Silhouette coefficient (**a**) and Davies–Bouldin index (**b**) as a function of the number of clusters *k*. Both indices support *k* = 2.

Figure 3 represents Silhouette coefficients and the Davies–Bouldin index against all *k*. The Silhouette coefficient (0.379) and the Davies–Bouldin index (1.03) both supported *k* = 2, and the two clusters were separated along the PC1 axis. Because all 28 coefficients of PC1 were positive, a larger PC1 score meant greater joint-angle SDs across the whole lower limb. Accordingly, the profile with the smaller mean PC1 score was designated the low-variability profile (*n* = 157), and the profile with the larger mean PC1 score was designated the high-variability profile (*n* = 113).

**Figure 4:**
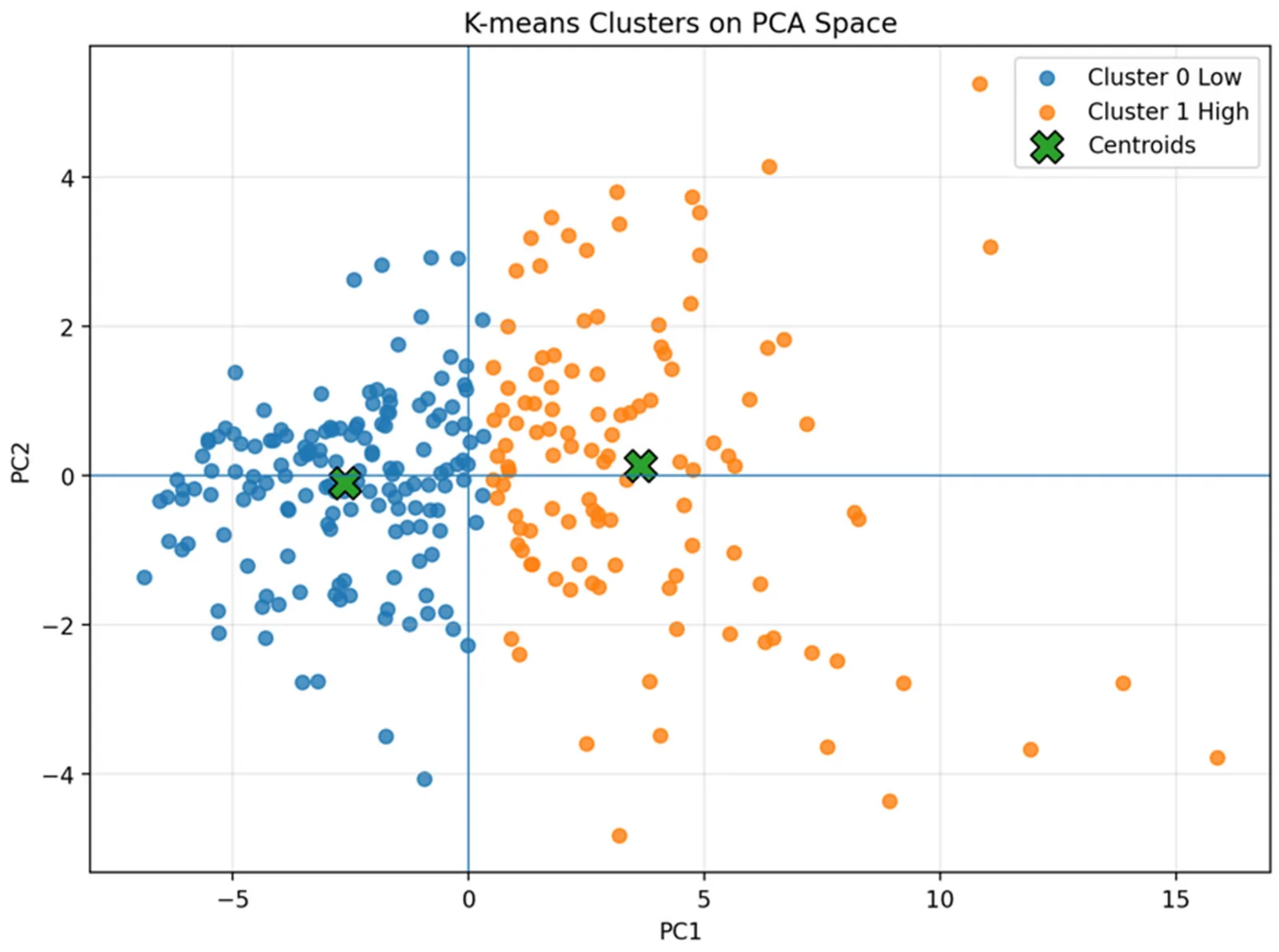
*k*-Means clusters in the PCA space (PC1–PC2).

The two profiles were clearly separated along the PC1 axis, whereas along the PC2 axis, the centroids of the two clusters lay at almost the same height (Figure 4). This is consistent with the Silhouette coefficient and the Davies–Bouldin index both supporting *k* = 2, and shows that the *k*-means partition was effectively determined by the single PC1 axis.

The proportion of high-variability trials increased monotonically with roll amplitude. The high-variability proportion was 0% at 0°, 0% at 5°, 28.3% at 10°, 68.3% at 15°, and 91.7% at 20° (Table 5). No trials were classified as high-variability up to 5°; high-variability trials first appeared at 10° (28.3%), and the proportion exceeded half at 15° (68.3%).

Within the same roll amplitude, the difference in the high-variability proportion between slow and fast was at most 3.3 percentage points—corresponding to 1 out of the 30 trials—for every amplitude. The direction of the difference was also inconsistent: at 10°, fast was greater than slow, whereas at 15° and 20°, slow was greater than fast.

The condition-wise distribution above showed that the high-variability proportion increased with roll amplitude but changed little with roll period. To quantify this response while accounting for the within-participant correlation, a GEE logistic regression was performed. The main effect of amplitude (Equation (1a)), the effect of period adjusted for amplitude (Equation (2)), and the interaction of the two variables (Equation (3)) were each fitted; the results are shown in Table 6.

The GEE analyses consistently showed a positive association between roll amplitude and membership in the operational high-variability profile (Table 6). All models accounted for repeated observations within participants (*n* = 30) using an exchangeable working correlation structure and robust sandwich standard errors. In Equation (1a), roll amplitude was significantly associated with profile membership (*p* < 0.001), with each 1° increase corresponding to an approximately 48% increase in the odds of membership.

Equations (1b) and (2) were fitted to the same 240 trials to compare the amplitude-only model with the model additionally adjusted for roll period. No statistically significant association between roll period and high-variability membership was detected (OR = 0.93, 95% CI 0.66–1.32, *p* = 0.704). The amplitude coefficient changed only minimally from β = 0.3912 in Equation (1b) to β = 0.3913 in Equation (2), suggesting no material evidence that adjustment for roll period confounded the estimated association between roll amplitude and high-variability membership. In Equation (3), after roll amplitude was centered at 10°, the fast-versus-slow contrast at 10° was not statistically significant (OR = 1.08, 95% CI 0.78–1.52, *p* = 0.636). The roll amplitude × roll period interaction was also not statistically significant (OR = 0.95, 95% CI 0.85–1.06, *p* = 0.373).

### 3.3. HDBSCAN

The grid search maximizing DBCV selected *min_cluster_size* = 6, *min_samples* = 2, and *cluster_selection_epsilon* = 0.0, yielding four clusters and outliers (DBCV = 0.061). The cluster structure was dominated by a single dense cluster (cluster 3, *n* = 139, 51.5%) and a large set of unassigned observations (outliers, *n* = 106, 39.3%), while the remaining three clusters together contained only 9.3% (25 trials) of the data (Table 7). In HDBSCAN, outliers do not constitute a separate cluster but denote observations not connected to the dense structure at a given density level. The result therefore reflected a structure in which one major dense region and the surrounding low-density observations predominate, rather than a structure with many stable subclusters.

#### 3.3.1. HDBSCAN Cluster Profiles and Density-Based Structure

**Figure 5:**
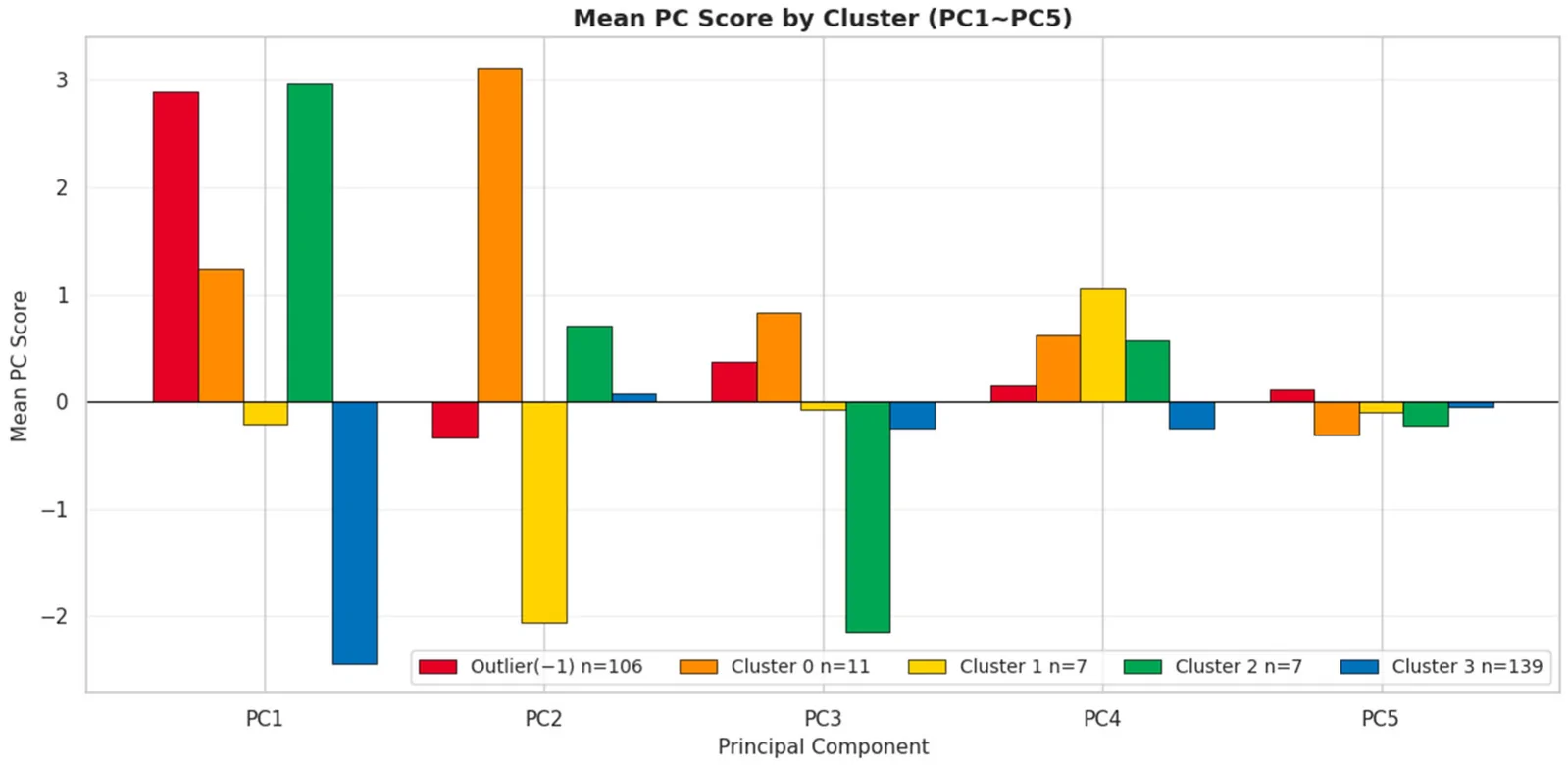
Mean PC score of each cluster (PC1–PC5).

Comparing the mean PC scores of the core cluster and the outliers showed that the two groups were separated mainly along PC1. The mean PC1 score of the outlier group was in the positive direction and that of the core cluster in the negative direction, whereas the differences on PC2–PC5 were smaller (Figure 5).

That is, the outlier status of an individual trial was associated mainly with the PC1 score, which coincided with the main split direction observed for *k*-means. However, because both analyses used the same PCA representation as input and PC1 explained 53.4% of the total variance, the large variance of PC1 dominated the Euclidean distance structure. This agreement was therefore interpreted not as an independent structural validation of one method by the other, but as a reconfirmation that PC1 was the main direction of variation in the data.

The DBCV was 0.061, close to 0, indicating weak support for a clearly separated density-based cluster structure. This was an expected result given the structure of the data. Because PC1 alone explained 53.4% of the total variance while each subsequent component explained less than 10%, the point cloud in the five-dimensional PC space was close to a one-dimensional shape elongated along PC1. Such a structure did not form clearly separated density peaks. The *k*-means result likewise showed no clear empty gap between the two profiles, which reflects the same continuous distribution.

Therefore, the low- and high-variability profiles produced by *k*-means are better interpreted not as two naturally and completely separated clusters, but as a practical dichotomization of a continuous variability distribution formed along PC1. The HDBSCAN outliers likewise do not denote an independent cluster; in the present data, they were distributed mainly toward larger PC1 scores and thus represent low-density observations departing from the main dense region.

#### 3.3.2. Inter-Individual Response Heterogeneity

The 106 trials labeled as outliers were not evenly distributed across the 30 participants. Although an average of 3.5 per participant (106/30) would be expected, the actual number of outlier trials ranged from 0 to 8. Outlier rates ranged from 0 to 0.89 across participants. For descriptive summarization only, participants with an outlier rate greater than 0.50 were labeled as the frequent-outlier group (*n* = 12), whereas the remaining participants were classified as the comparison group (*n* = 18). This operational cutoff was not intended to define a clinical or safety-related threshold.

S12 had the greatest outlier rate (8 out of 9 trials), followed by S09 (7 out of 9 trials). In contrast, S15, S20, S23, and S29 remained within the core density structure in all nine trials, including the 20° condition. These participant-level differences are presented as descriptive comparisons; because the condition-specific outlier probability differed and a within-participant repeated-measures structure was present, no significance testing was performed for individual participants.

The conditions under which outliers occurred were also spread over a wider range than the group-level response. The outlier rate by amplitude was 3.3% at 0°, 11.7% at 5°, 41.7% at 10°, 50.0% at 15°, and 71.7% at 20°. Unlike the *k*-means high-variability profile, which first appeared at the 10° condition (Section 3.2), low-density observations were also found at the 0° and 5° conditions. The eight outlier trials that occurred in the low-amplitude (0–5°) conditions came from five participants (S09, S12, S19, S22, S27), of whom S22 was the only case that departed under the no-roll 0° condition. However, because the HDBSCAN outlier status and the *k*-means profile membership are indices defined under different cluster criteria, they were not interpreted as the temporal ordering of the same state transition.

The nine trial-level scores for each retained PC were averaged within each participant to characterize the PC-space positions associated with repeated outlier labeling. The resulting participant-level mean scores were then compared descriptively between participants with at least five outlier trials (frequent-outlier group; *n* = 12) and the remaining participants (comparison group; *n* = 18), with the standardized group differences summarized using Cohen’s *d* (Table 8).

Based on the predefined descriptive benchmarks, the absolute standardized differences were large for PC1 (*d* = 0.962), PC2 (*d* = −0.842), and PC3 (*d* = 0.839). The difference for PC5 (*d* = 0.797) fell just below the conventional large-effect benchmark of 0.80, whereas the difference for PC4 (*d* = −0.353) was small. Thus, the frequent-outlier and comparison groups showed substantial descriptive differences along PC1, PC2, PC3, and PC5 rather than along PC1 alone. This pattern suggests that repeated density-based departure may involve multiple dimensions of the retained PC space rather than overall variability magnitude alone. However, because PC2–PC5 showed lower bootstrap stability, these secondary-component differences were interpreted as exploratory.

The individual profiles likewise showed that frequent-outlier labeling was not invariably associated with an extreme PC1 score (Table 9). For each PC, the nine trial-level scores were averaged within each participant, and the resulting participant-level means were standardized using the mean and sample standard deviation across all 30 participants. A z-score of 0 therefore represents the overall participant mean, whereas a larger absolute z-score indicates a more extreme position within the sample. S12, the participant with the greatest number of outlier trials, had a PC1 z-score of 0.147, which was close to the overall mean, but a PC5 z-score of 2.694. Similarly, S04 had a PC1 z-score of 0.020 but a PC4 z-score of −2.149. These cases indicate that repeated HDBSCAN outlier labeling could occur even when the participant’s mean PC1 score was close to the sample average and may instead be associated with an extreme position along a secondary component.

## 4. Discussion

Walking on a rolling support surface challenges multijoint gait control and has practical implications for safe mobility in shipboard environments [1,2]. However, previous studies have primarily reported joint-specific group averages [7], leaving unclear how roll-related gait variability is organized across the lower limb and how individual responses differ from the dominant population-level pattern. Clarifying both the shared multijoint structure and participant-level heterogeneity may provide a biomechanical basis for future individualized assessment of mobility responses and fall susceptibility in maritime settings. A multivariate approach capable of integrating correlated joint responses while distinguishing population-level patterns from individual departures was therefore needed. The primary purpose of this study was to determine how gait variability under simulated ship-roll conditions was organized across multiple lower-limb joints and whether this response differed across participants. Specifically, we sought to identify the low-dimensional structure of multijoint gait variability, quantify the associations of roll amplitude and period with membership in an operational high-variability profile, and characterize participant-level departures from the dominant multivariate response.

This study reanalyzed lower-limb joint-angle variability data collected under simulated ship roll conditions using principal component analysis and two types of cluster analysis, jointly quantifying the group-level gait variability response to roll amplitude and the inter-individual heterogeneity underlying it. The main findings are summarized in four points. First, PC1 accounted for the largest share of the total variance (53.4%) and summarized the primary shared pattern across the 28 multijoint variability features, while additional variation remained across the secondary components. Second, along this axis the trials were divided into two profiles—low-variability and high-variability—and profile membership was strongly associated with roll amplitude (OR = 1.48 per 1° increment). Third, after adjusting for roll amplitude, roll period (6 s vs. 12 s) provided no significant additional information. Fourth, the group-level amplitude effect masked substantial inter-individual heterogeneity.

### 4.1. Primary Shared Pattern of Multijoint Variability

PC1 accounted for 53.4% of the total variance, substantially more than PC2 (8.5%), and therefore represented the primary shared pattern of multijoint variability. Although additional variation remained across the secondary components, all 28 PC1 coefficients had the same sign, with the largest contributions arising from sagittal-plane variability of the bilateral knees, ankles, and hips. This loading pattern suggested coordinated covariation across multiple lower-limb joints rather than variability concentrated in a single joint. The participant-level bootstrap further showed that the PC1 loading pattern was highly reproducible within the present sample.

This multijoint pattern was consistent with the joint-specific trends previously reported from the same dataset. The previous analysis showed that the SDs of the peak joint angles increased with roll amplitude across the lower-limb joints, with particularly large effect sizes for knee flexion, hip flexion, and plantarflexion [7]. PC1 integrated the common variation across these joint-specific measures into a single summary dimension, while the secondary components represented additional aspects of the variability structure.

The shared covariation may reflect active postural reorganization, reduced inter-cycle reproducibility, or a combination of both. Although the present data cannot distinguish between these mechanisms, the coordinated loading pattern suggested that the response involved multiple lower-limb joints rather than an isolated joint-specific change.

### 4.2. Roll-Amplitude-Related Increase in High-Variability Profile Membership

The proportion of trials assigned to the high-variability profile increased progressively with roll amplitude: 0% at 0° and 5°, 28.3% at 10°, 68.3% at 15°, and 91.7% at 20°. Consistent with the logistic regression results, larger roll amplitude was associated with a greater likelihood of high-variability profile membership. The condition-specific proportions demonstrated a graded shift toward the high-variability profile, with the largest observed increase occurring between 10° and 15°. However, these values represent descriptive changes across the tested conditions rather than formally established thresholds.

The increasing prevalence of the high-variability profile at larger amplitudes may reflect greater challenges to postural control and lower-limb coordination under stronger roll perturbations. Accordingly, these findings can provide a quantitative basis for grading amplitude-related gait disturbance and support future investigations into whether such changes predict loss of balance, near-falls, or falls. Because fall-related outcomes were not measured, the present findings represent evidence of amplitude-related changes in gait variability rather than direct evidence of fall risk. Together with the previously reported sharp increase in joint variability at 20° [7], these results could help identify roll-amplitude ranges that warrant further investigation when developing evidence-based criteria for onboard gait safety.

### 4.3. No Detected Effect of Roll Period

After adjusting for roll amplitude, no statistically significant association between roll period and high-variability membership was detected (OR = 0.93, 95% CI 0.66–1.32, *p* = 0.704). However, the confidence interval encompassed potentially meaningful decreases and increases in the odds, and the result therefore does not establish the absence of a roll-period effect or sufficient statistical power to exclude such an effect. Descriptively, the difference in the high-variability proportion between the slow and fast conditions was only 3.3 percentage points, corresponding to 1 out of 30 trials, at both 10° and 15°, and the direction of the difference was inconsistent across amplitudes. These findings indicate that no evidence of a roll-period effect was detected within the tested 6 s and 12 s conditions.

This finding does not imply that the temporal characteristics of ship motion are irrelevant. The 6 s and 12 s periods evaluated here were both markedly longer than a typical gait cycle of approximately 1 s and may therefore have produced similar, quasi-static changes in support-surface inclination from the perspective of human balance control. Different responses may occur at other roll periods, particularly at shorter periods closer to the natural frequencies of postural sway or gait. Accordingly, the present findings should be limited to the two period conditions examined. A previous study also identified the systematic investigation of roll period as an area for future research [6], and the present study provides initial evidence that no statistically significant difference was detected between the tested 6 s and 12 s conditions.

### 4.4. Association Between Roll Amplitude and High-Variability Membership

Roll amplitude showed a strong positive association with membership in the high-variability profile. Each 1° increase in roll amplitude was associated with an approximately 1.48-fold increase in the odds of high-variability membership. Because the *k*-means profiles were separated primarily along PC1, this association indicated that increasing roll amplitude progressively increased the likelihood of a coordinated elevation in variability across the lower-limb joints, particularly in sagittal-plane movements of the bilateral knees, ankles, and hips. This multivariate pattern is consistent with the previous joint-by-joint analysis of the same experimental data, which showed amplitude-related increases in joint-angle variability across the lower limbs, with particularly large effects in sagittal-plane variables [7]. The present analysis further shows that these joint-specific changes can be represented as a common whole-limb variability profile.

The observed proportion of high-variability trials increased from 0% at 0° and 5° to 28.3% at 10°, 68.3% at 15°, and 91.7% at 20°. Together with the GEE results, these condition-specific proportions demonstrate a consistent amplitude-dependent increase in high-variability membership at the group level. However, the participant-level results indicate that sensitivity to increasing roll amplitude differed considerably among individuals.

From a safety perspective, the coordinated increase in whole-limb variability may reflect greater demands on postural control as roll amplitude increases and may therefore be relevant to understanding movement responses associated with shipboard falls. Nevertheless, increased variability may also represent an adaptive response to the moving support surface, and the present study did not measure falls, near-falls, balance loss, handrail contact, or other external indicators of instability. High-variability membership should therefore be interpreted as a measure of the movement response to roll motion rather than as a direct indicator of fall risk. Further studies should determine whether this variability profile is associated with actual safety and performance outcomes in real or more ecologically valid maritime environments.

### 4.5. Methodological Implications

This study showed that multi-joint lower-limb variability can be expressed as a primary low-dimensional index (PC1) and a binary profile. Such a representation might be used in the design of wearable-sensor-based systems for monitoring gait instability. An approach that computes a single composite variability index instead of simultaneously tracking many channels of individual joints can reduce the computational and sensor burden in real-time field applications. At the same time, the existence of inter-individual heterogeneity warns that such a system, if operated on a group criterion alone, may miss vulnerable individuals, and it suggests the possibility of individual screening using short, low-intensity roll exposure.

### 4.6. Limitations

This study has several limitations. First, the participants were 30 healthy adults, who may differ from experienced seafarers adapted to ship motion through long-term shipboard experience. The present results should be understood as reflecting the initial adaptation response of individuals unaccustomed to roll.

Second, the inter-individual heterogeneity analysis relied on an exploratory procedure of the HDBSCAN outlier rate and post hoc PC and raw-feature comparisons. Because the outlier group was defined by cluster labels derived in the PC space, the PC comparison in the same space (Table 8) is circular in nature and cannot be regarded as an independent hypothesis test. A linear mixed model with PC1 as the outcome variable and participant-specific random intercepts and random slopes would be more appropriate for directly modeling participant-specific roll sensitivity, and this remains a task for future work.

Third, the 270 trials comprised nine repeated observations from 30 participants and therefore should not be considered statistically independent observations when evaluating PCA stability. Participant-level bootstrap analysis showed that the dominant PC1 structure was highly stable, whereas PC2–PC5 showed smaller and more variable congruence. Accordingly, interpretation of PC1 as the dominant multijoint variability axis is supported within the present sample, whereas the joint- and plane-specific interpretations of the secondary components require caution and validation in an independent sample.

Fourth, and most importantly, external proxy indicators of fall risk—such as the margin of stability, handrail contact, and near-fall events—were not available. The observed increase in variability and the inter-individual departures were therefore interpreted only as heterogeneity of the movement response and not as fall risk, and the relationship between this response and actual fall risk must be verified further in real maritime environments.

## 5. Conclusions

This study showed that under simulated ship-roll conditions, PC1 accounted for the largest share of lower-limb joint-angle variability and summarized the primary shared multijoint pattern, while additional variability remained outside PC1. The k-means analysis of the retained PC1–PC5 scores divided the gait trials into a low-variability profile (*n* = 157) and a high-variability profile (*n* = 113), with the two profiles separated primarily along PC1. Roll amplitude was strongly associated with membership in the high-variability profile. For each 1° increase in roll amplitude, the odds of belonging to the high-variability profile increased approximately 1.48-fold (*p* < 0.001). In contrast, after adjustment for roll amplitude, no statistically significant difference in high-variability membership was detected between the tested 6 s and 12 s conditions (OR = 0.93, 95% CI 0.66–1.32, *p* = 0.704).

The density-based analysis suggests that these two profiles are not naturally separated into distinct clusters but a dichotomization of a continuous distribution formed along PC1 (DBCV = 0.061). Furthermore, the group-level and individual-level responses did not fully coincide. The group high-variability profile first appeared at 10°, but some participants had already departed at the 0° and 5° conditions, whereas others did not depart even at the maximum amplitude. Exploratory participant-level differences were also observed across the secondary components; however, because PC2–PC5 showed reduced bootstrap stability, firm conclusions regarding the joint- and plane-specific characteristics underlying this heterogeneity cannot be drawn.

In summary, by jointly quantifying the change in gait variability profiles with roll amplitude and the heterogeneity of individual responses, this study’s results provide baseline data for future investigation of task- and gait-limitation criteria across different levels of ship motion. The representation of multi-joint lower-limb variability as a low-dimensional index and a binary profile can be used in the development of wearable-sensor-based gait-instability monitoring systems. However, because falls, near-falls, task interruption, and real-world shipboard performance were not measured, the present results should not be interpreted as direct evidence of fall risk or as a basis for operational safety thresholds. Future work should validate multijoint variability measures against external safety and performance outcomes in seafarers and in real or more ecologically valid maritime environments.

## Figures and Tables

**Table 1:** Experimental conditions. Each roll amplitude was tested under a slow (12 s period) and a fast (6 s period) roll condition.

Trial	Degrees of Rolling	Speed
1	0°	—
2	5°	slow
3	5°	fast
4	10°	slow
5	10°	fast
6	15°	slow
7	15°	fast
8	20°	slow
9	20°	fast

**Table 2:** Explained variance ratio of the retained principal components.

Principal Component	Explained Variance Ratio
PC1	0.534
PC2	0.085
PC3	0.071
PC4	0.052
PC5	0.039

**Table 3:** The ten features with the largest PC1 coefficients.

Feature	PC1 Coefficient
knee_angle_l_max_sd	0.234
knee_angle_r_max_sd	0.232
ankle_angle_r_max_sd	0.229
hip_flexion_r_max_sd	0.229
ankle_angle_l_max_sd	0.225
ankle_angle_r_min_sd	0.222
ankle_angle_l_min_sd	0.221
knee_angle_r_min_sd	0.220
hip_flexion_l_max_sd	0.219
knee_angle_l_min_sd	0.217

**Table 4:** Participant-level bootstrap stability of the retained principal components.

PC	Bootstrap EVR, Median (2.5th–97.5th Percentile)	Median Absolute Tucker Congruence (2.5th–97.5th Percentile)
PC1	0.5420 (0.4979–0.5832)	0.9971 (0.9917–0.9991)
PC2	0.0959 (0.0792–0.1217)	0.8592 (0.5921–0.9748)
PC3	0.0731 (0.0606–0.0868)	0.7997 (0.5239–0.9569)
PC4	0.0547 (0.0444–0.0671)	0.7540 (0.3509–0.9286)
PC5	0.0419 (0.0338–0.0517)	0.6656 (0.0938–0.9069)

**Table 5:** Proportion of high-variability trials by roll amplitude.

Roll Amplitude	High-Variability Proportion (%)
0°	0
5°	0
10°	28.3
15°	68.3
20°	91.7

**Table 6:** GEE logistic regression results.

Model	*n*	Term	*β* (SE)	OR (95% CI)	*p*
Equation (1a)	270	Roll amplitude (1°)	0.3922 (0.0524)	1.48 (1.34–1.64)	<0.001
Equation (1b)	240	Roll amplitude (1°)	0.3912 (0.0539)	1.48 (1.33–1.64)	<0.001
Equation (2)	240	Roll amplitude (1°)	0.3913 (0.0538)	1.48 (1.33–1.64)	<0.001
		Roll period	−0.0676 (0.177)	0.93 (0.66–1.32)	0.704
Equation (3)	240	Roll amplitude (1°)	0.4190 (0.0661)	1.52 (1.34–1.73)	<0.001
		Roll period	0.0811 (0.1714)	1.08 (0.78–1.52)	0.636
		Amplitude × period	−0.0521 (0.0585)	0.95 (0.85–1.06)	0.373

**Table 7:** HDBSCAN cluster sizes (−1 denotes outliers).

Cluster	*n*	%
3	139	51.5
−1	106	39.3
0	11	4.1
1	7	2.6
2	7	2.6

**Table 8:** Mean PC scores of the frequent-outlier group and the comparison group and the corresponding Cohen’s *d*.

PC	Frequent-Outlier Group (*n* = 12)	Comparison Group (*n* = 18)	Cohen’s *d*
PC1	1.013	−0.675	0.962
PC2	−0.640	0.427	−0.842
PC3	0.578	−0.385	0.839
PC5	0.336	−0.224	0.797
PC4	−0.223	0.149	−0.353

**Table 9:** Outlier counts and PC z-scores of the frequent-outlier participants.

Subject	n_Outlier	PC1_z	PC2_z	PC3_z	PC4_z	PC5_z
S12	8	0.147	−0.745	−0.486	1.445	2.694
S09	7	0.770	−0.066	1.258	0.896	−0.052
S02	6	1.792	−1.104	−1.562	−0.441	0.112
S04	6	0.020	0.535	−0.293	−2.149	1.527
S06	6	−1.115	−0.964	1.530	0.925	−0.592
S07	6	2.162	−1.243	−1.055	−1.586	0.193
S26	6	1.490	1.477	2.173	0.289	1.554
S30	6	1.420	−0.236	0.138	−0.400	1.489
S03	5	1.080	−1.141	1.143	−1.595	−1.054
S05	5	0.024	−0.614	1.838	−0.927	0.579
S13	5	−2.061	−1.830	1.191	2.132	0.493
S22	5	0.606	0.256	−0.216	−1.138	−1.527

## Data Availability

The data presented in this study are available on request from the corresponding author due to privacy and ethical restrictions.
